# Knowledge of Physical Activity Guidelines and Its Association with Meeting Physical Activity and Sedentary Behavior Recommendations in Adolescents

**DOI:** 10.3390/children12081084

**Published:** 2025-08-19

**Authors:** André de Araújo Pinto, Guilherme José Silva Ribeiro, Andreia Pelegrini

**Affiliations:** 1 Health Sciences Center, State University of Roraima, Boa Vista 69306-530, Roraima, Brazil; 2 Graduate Program in Nutrition Science, Department of Nutrition, Federal University of Viçosa, Viçosa 36570-900, Minas Gerais, Brazil; 3 Study and Research Group in Kinanthropometry, Center of Health and Sports Sciences, Santa Catarina State University, Florianópolis 88080-350, Santa Catarina, Brazil

**Keywords:** adolescents, exercise, guidelines, health literacy, sitting time

## Abstract

**Highlights:**

**What are the main findings?**

• Adolescents show low knowledge of official physical activity recommendations, particularly regarding the recommended frequency, duration, and intensity.
• Greater awareness of the guidelines is associated with higher physical activity levels and less sedentary behavior, regardless of sex.

**What is the implication of the main finding?**

• Expanding the dissemination of official physical activity recommendations may foster healthier and more active lifestyle choices among adolescents.
• Health promotion strategies should integrate physical activity guideline content into school and family environments, using accessible and contextually relevant approaches.

**Abstract:**

Background/Objectives: Despite global efforts to promote moderate-to-vigorous physical activity (MVPA) among youth, limited evidence exists regarding adolescents’ knowledge of official physical activity (PA) guidelines. The aim of this study was to assess adolescents’ knowledge of MVPA guidelines and examine its potential association with meeting PA recommendations and levels of sedentary behavior. Methods: This cross-sectional study was conducted in 2025 with a sample of 1032 adolescents (50.5% boys) from northernmost Brazil. Data were collected using a self-administered questionnaire and included information on knowledge of PA guidelines, PA levels, sedentary behavior duration, and sociodemographic characteristics. Associations were tested using logistic regression models. Results: Only 11.7% of adolescents accurately identified all components of the PA recommendations. Boys who met PA guidelines were 15.76 times more likely to be aware of the official recommendations (95% CI: 7.14–24.48), while girls had 10.05 times higher odds (95% CI: 4.43–16.67). Adolescents who were less sedentary (<3 h/day) were significantly more likely to know the guidelines, both among boys (OR = 2.00; 95% CI: 1.08–3.70) and girls (OR = 3.13; 95% CI: 1.12–8.33). Conclusions: The low level of awareness regarding official PA guidelines among adolescents is concerning, particularly given the strong association between such knowledge and the adoption of more active and less sedentary behaviors. Public health and educational strategies should prioritize health literacy in school curricula and community-based programs to promote more active lifestyles and reduce sedentary behavior among youth. Future studies should use longitudinal designs to clarify causal links and test practical interventions.

## 1. Introduction

Regular physical activity (PA) improves physical, mental, and social health, helps prevent chronic diseases, and supports self-esteem and academic performance in adolescents [1]. Therefore, it is particularly concerning that 84% of youth worldwide fail to meet the World Health Organization (WHO) recommendation of at least 60 min/day of moderate-to-vigorous physical activity (MVPA) [2]. In response to this alarming trend, the WHO updated its global guidelines and launched an action plan to reverse this trend by 2030, emphasizing public policy, active school environments, and healthy lifestyle promotion [3].

Failure to meet PA recommendations also affects 84.7% of adolescents in low- and middle-income countries [4]. In Brazil, the prevalence of insufficient PA reached 82% in 2019 (<300 min/week) [5], with evidence suggesting a 29% increase in recent years [6]. Aligned with global efforts [3], the Brazilian Ministry of Health has implemented national strategies to address this issue, including the release of the *Physical Activity Guidelines for the Brazilian Population* in 2021 [7]. Given the persistently high physical inactivity rates among adolescents [2,4,5] and limited evidence from northern Brazil, we hypothesize that most remain unaware of official PA recommendations.

In adults, awareness of PA guidelines is linked to greater engagement in PA [8,9]. Among adolescents, findings are inconsistent, ranging from positive associations [10] to no significant relationships [11]. This gap is relevant because knowledge of PA recommendations is a core component of health literacy. Health literacy is defined as the ability to access, understand, evaluate, and apply health-related information to make informed daily decisions [12]. Physically active adolescents are more likely to encounter health information [13] and, consequently, be familiar with official PA guidelines. However, cross-country inconsistencies, lack of sex-specific analyses, and absence of representative studies in parts of Brazil highlight the need for regional investigations. This study examines the link between knowledge of PA guidelines and adherence among Brazilian adolescents, providing evidence to guide future health interventions.

Alongside low PA levels, sedentary behavior in adolescents is linked to adverse health outcomes, including cardiovascular disease and higher mortality risk [14], and is increasing among Brazilian youth [5]. An estimated 54% of Brazilian adolescents spend over three hours a day sitting [5], contrary to national [7] and international [3] recommendations to reduce sedentary time. Physically active adolescents are typically more exposed to health-promoting environments [8,15], whereas highly sedentary peers may be less inclined to make informed health choices [9].

Understanding adolescents’ knowledge of PA recommendations is necessary to guide the development of more effective public health messaging. Practical knowledge about the minimum recommended duration, frequency, and intensity of PA may increase adolescents’ awareness of its health benefits and empower them to make more informed choices [8,9]. Moreover, mapping adolescents’ familiarity with these guidelines may reveal disparities in access to health information and help tailor interventions to local contexts. Therefore, the present study aimed to assess adolescents’ knowledge of PA guidelines and examine its association with meeting recommended PA levels and sedentary behavior. We hypothesize that knowledge is generally low and that greater awareness is linked to higher PA engagement and reduced sedentary time.

## 2. Materials and Methods

### 2.1. Study Design and Location

This school-based cross-sectional study is part of the larger project “Health Risk Behaviors Among Adolescents in Boa Vista, Roraima: The School Health Questionnaire”. Data collection took place between March and May 2025 in Boa Vista, the capital of Roraima, Brazil, which has a population of 413,486 inhabitants, including approximately 35,300 adolescents aged 15 to 19 years, according to the 2022 national census [16]. The city is the main urban center in the state, housing 65% of its total population.

### 2.2. Population and Sample

According to the 2024 school census, there were 28,745 high school students enrolled across 58 schools in the city, distributed among nine educational districts. The sample size was calculated based on an expected prevalence of 50% (to maximize sample size), a 95% confidence level, a 5% margin of error, and a design effect (deff) of 2. An additional 10% was added to account for potential nonresponse or missing data. These parameters yielded a minimum required sample of 834 adolescents. The inclusion criteria were students of both sexes, aged 14 to 19 years, enrolled in high school, and present in the classroom on the day of data collection. The exclusion criteria were students outside the defined age range, those with physical or clinical conditions preventing participation, and pregnant adolescents.

A two-stage stratified cluster sampling design was employed. In the first stage, the target population was stratified by the nine educational districts, as defined by the State Department of Education. The estimated minimum sample was then proportionally allocated to each district according to the percentage of enrolled students. Within each district, the largest school (in terms of student population) was selected. In the second stage, classrooms within each selected school were randomly chosen until the proportional number of adolescents was reached. When necessary, more than one classroom per grade level was selected to meet sample size requirements.

All students in selected classrooms were invited, ensuring full inclusion of each cluster. This process resulted in an initial sample of 1051 adolescents. Nineteen were excluded: one over the age limit (>19 years), six pregnant, and twelve unable to complete the questionnaire independently or whose responses were completed by teachers. The final sample included 1032 adolescents.

### 2.3. Study Variables

Data were collected using a self-administered, closed-ended questionnaire, pilot-tested with 67 adolescents (35 girls) from a public school not in the main study. The test–retest interval was three days. Knowledge of PA guidelines (dependent variable) was measured using an instrument adapted from Marques et al. [10], with three multiple-choice questions on PA frequency, duration, and intensity, based on current adolescent health recommendations [3]. The three questions demonstrated moderate agreement (Kappa = 0.43–0.63; *p* < 0.001) [17].

The questions included the following:

(a) Frequency: “*On how many days per week, at a minimum, should physical activity be practiced to achieve general health benefits?*” with options ranging from one to seven days; the correct answer was “seven”.
(b) Duration: “*On the days when physical activity is practiced, how much time, at a minimum, should adolescents be physically active to stay healthy?*” with seven options ranging from “15 min/day or more” to “105 min/day or more” in 15-min increments; the correct answer was “60 min/day or more”.
(c) Intensity: “*During physical activity sessions, what is the recommended intensity to maintain or improve health?*” with four options: light, moderate, vigorous, and moderate to vigorous; the correct answer was “moderate to vigorous”.
(d) Adolescents who correctly answered all three questions were categorized as “knowledgeable”, while those who missed one or more were categorized as “not knowledgeable”.

Adherence to PA guidelines was assessed using the short version of the International Physical Activity Questionnaire (IPAQ), adapted for Brazilian adolescents [18] and widely used in national surveys [6,19,20]. The instrument was culturally adapted and validated for Brazilian adolescents [18]. The questionnaire covered a typical week, collecting MVPA frequency and duration across leisure, commuting, household chores, and work. Participants reported the number of days and the average time spent on each activity. Adolescents were classified as “meeting recommendations” if they engaged in ≥60 min/day of MVPA, per WHO [3] and Brazilian PA Guidelines [7]. These measures demonstrated moderate to good reproducibility [21], with intraclass correlation coefficients (ICC) ranging from 0.66 to 0.90 for frequency and duration of moderate and vigorous activities (*p* < 0.001).

Sedentary behavior (h/day) was measured using two IPAQ items on sitting time [18]. Average sitting time was calculated as [(weekday sitting time × 5) + (weekend sitting time × 2)]/7. For analysis, sedentary time was dichotomized as <3 h/day (reference) vs. ≥3 h/day [2,22,23]. The ICCs were 0.71 for weekdays and 0.67 for weekends, indicating moderate reliability (*p* < 0.001) [21].

Sociodemographic characteristics were included as covariates and encompassed: age (continuous, in complete years), sex (male or female), self-reported race/ethnicity (White, Black, Brown [mixed-race], Yellow, and Indigenous), and monthly household income (≤2 minimum wages; 3–5 minimum wages; ≥6 minimum wages). Adolescents also self-reported the educational level of the head of household (no formal education, elementary school, high school, or higher education) and the identity of the head of household (father, mother, or other).

### 2.4. Statistical Analysis

Descriptive analyses were performed for all variables. Means and standard deviations were calculated for continuous variables (e.g., age), while absolute and relative frequencies were reported for categorical variables. The association between sex and knowledge of MVPA guidelines was assessed using Pearson’s chi-square test. Binary logistic regression estimated odds ratios (ORs) and 95% confidence intervals (95% CI) for PA guideline knowledge by PA adherence and sedentary behavior. Analyses were stratified by sex and adjusted for age, household income, race, and head of household’s education, accounting for known biological and sociocultural differences in PA patterns and health literacy [13,20]. Model fit was evaluated using Nagelkerke’s pseudo-R^2^. Cases with missing data were excluded listwise, as missing values were <1% and did not affect statistical power. All statistical procedures were performed using IBM SPSS Statistics software (version 20.0). A significance level of 5% (*p* < 0.05) was adopted for all analyses.

## 3. Results

Table 1 shows the characteristics of the 1032 adolescents, stratified by sex (521 boys, 511 girls). Mean age was 16.1 years (±0.9), with no sex difference. Most (53.8%) identified as mixed race, more common among girls (55.8%). Overall, 59.5% lived in households earning ≤2 minimum wages. Among higher-income households, boys were more frequent (14.0%) than girls (7.0%). Fathers were reported as household heads by 42.4% and mothers by 57.6%. Eight in ten household heads had at least a secondary education. Only 40.5% met the ≥60 min/day MVPA recommendation; boys were more active (55.5%). Sedentary behavior ≥3 h/day was highly prevalent (88.9%), more common among girls (94.7%).

**Figure 1:**
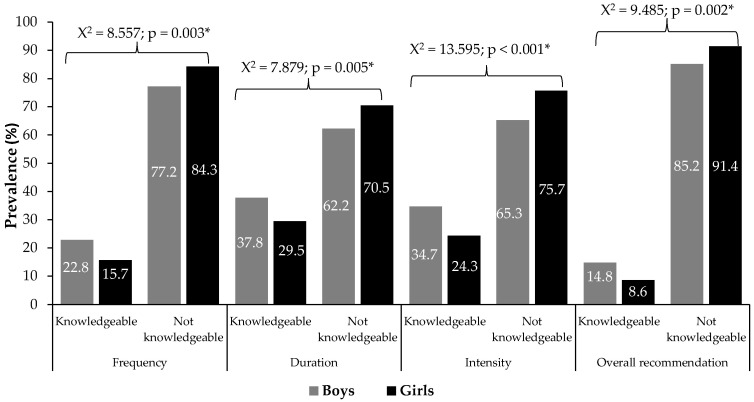
Knowledge of specific and general moderate-to-vigorous physical activity (MVPA) guidelines among adolescents, by sex. Note: Values expressed as percentages (%). χ^2^: Pearson’s chi-square test value. * *p* < 0.05 indicates a statistically significant association between sex and knowledge of the guidelines.

Figure 1 shows PA guideline knowledge by sex. Only 11.7% correctly identified all PA recommendation components—about one in ten. Sex was significantly associated with knowledge of guidelines (*p* < 0.05), including frequency, duration, intensity, and overall recommendations, and in all domains, boys had greater PA guideline awareness than girls. These differences, though significant (*p* < 0.05), had small effect sizes (Cramer’s V: 0.087–0.115), indicating modest sex disparities.

Table 2 presents sex-stratified logistic regression results for PA adherence and sedentary behavior in relation to MVPA guideline knowledge. Boys who engaged in ≥60 min/day of MVPA were approximately 15 times more likely to know the guidelines (OR = 15.76; 95% CI: 7.14–24.48). Among girls, those who were active for ≥60 min/day were approximately 10 times more likely to know the recommendations (OR = 10.05; 95% CI: 4.43–16.67). Adolescents who were sedentary for <3 h/day were more likely to know the recommendations, with this association observed in both boys (OR = 2.00; 95% CI: 1.08–3.70) and girls (OR = 3.13; 95% CI: 1.12–8.33). Model fit (Nagelkerke’s pseudo-R^2^) was 0.347 for boys and 0.331 for girls.

## 4. Discussion

This study found low knowledge of MVPA guidelines among adolescents, with only one in ten correctly identifying all components. Meeting PA recommendations and spending less time sedentary were both linked to greater knowledge, independent of sociodemographic factors, and consistent across sexes. These results underscore the role of health literacy as a strategy to promote PA in schools and communities, equipping adolescents to make healthier choices.

With only 11.7% of adolescents aware of the official PA recommendations, it is reasonable to infer that the primary goals of Brazil’s PA Guidelines, which are to inform and encourage regular PA, are not effectively reaching this population. Adolescents have historically been characterized by high levels of physical inactivity, as previously reported in the literature [2,4,5]. This result is slightly better than that observed among Portuguese adolescents (3.6%) [10], but lower than findings reported in India (18.9%) [24] and especially in Ireland (67.0%) [25]. This situation may partly reflect the limited effectiveness of dissemination strategies, which often fail to adapt language and communication channels to the everyday realities and interests of adolescents [8,26]. Schools often lack PA health content, focusing physical education on technical or sport-specific skills [27,28]. At home, parents or guardians may also lack knowledge, limiting family discussion and reinforcement of these guidelines [29,30].

This study also revealed a positive association between meeting PA recommendations and greater knowledge of the guidelines. However, this relationship is complex, as previous studies have produced mixed and sometimes contradictory findings, indicating the need for further research [31]. For example, among Irish adolescents, knowledge of the guidelines did not increase the likelihood of meeting them. Surprisingly, an inverse association was observed among physically active boys, which challenges assumptions derived from behavior change theories [25]. Similarly, a study with Hungarian adolescents showed that although they demonstrated higher levels of health-related knowledge, this did not directly translate into higher levels of PA. These findings suggest that knowledge, although important, may not be sufficient to produce behavioral change [32]. These suggest that knowledge alone may not drive behavior. Conversely, studies with university students [9], French adolescents [33], and overweight adolescents in intervention programs [34] found that greater knowledge aligned with higher PA, indicating that context and population may influence this relationship.

Although knowledge of PA guidelines is expected to lead to higher levels of PA, this relationship is multifaceted and not always confirmed in practice [11]. This is because adolescent PA is influenced by multiple factors, including motivational and cognitive aspects, environmental barriers, and social support [35], with knowledge representing only one of these components [28]. It is believed that even when adolescents are aware of what is recommended, the ability to apply this knowledge in daily life depends on their capacity to interpret, value, and put it into practice [12]. Furthermore, behavior may also influence knowledge, with active youth more likely to embrace information that reinforces their habits [36]. Future research should use sensitive tools and integrated models to explore not only what adolescents know but how and in which contexts this translates into greater PA.

In this study, shorter durations of sedentary behavior were associated with higher odds of adolescents being knowledgeable about PA guidelines. Although this direct association is novel, the finding is consistent with existing literature that links low levels of knowledge to less healthy behaviors. For example, a study with Brazilian adolescents found that inaccurate knowledge of screen time recommendations was inversely associated with progression through the stages of behavior change, suggesting that lack of information hinders the adoption of healthier habits [36]. Similarly, another study showed that greater knowledge about PA-related injuries was associated with a reduced likelihood of such injuries occurring [37], reinforcing the protective role of knowledge against adverse outcomes. In addition, evidence indicates that lower levels of health literacy are associated with less healthy behaviors, such as lower levels of PA and higher consumption of sugar-sweetened beverages [12].

Several hypotheses may explain this association. First, more sedentary adolescents, particularly those whose sedentary time is associated with screen use, may have reduced exposure to school, family, and social contexts where PA guidelines are disseminated [36]. Second, for individuals with predominantly sedentary lifestyles, PA information may not be perceived as relevant or a priority, limiting their interest in and assimilation of these guidelines [10,35]. Third, the observed negative association may not reflect a direct causal relationship but rather distinct processes influenced by factors such as motivation, social support, and the surrounding environment [9]. Finally, poorly adapted messages, including those that are overly technical or disconnected from adolescents’ lived experiences, may be ignored or misunderstood, particularly by more sedentary youth. Further studies, including qualitative approaches, are needed to explore in greater depth the factors that influence the relationship between sedentary behavior and knowledge of PA guidelines, taking into account the diverse profiles of adolescents.

### 4.1. Practical Implications and Recommendations

The findings of this study highlight the pressing need to strengthen health literacy strategies targeting adolescents. Considering the limited awareness of PA guidelines and their positive association with healthier behaviors, schools should incorporate guideline-based content into physical education curricula, using clear language and interactive approaches. Training programs for teachers can help shift the emphasis from sport-specific skills toward broader health promotion. Beyond the school setting, engaging families and implementing community-based initiatives, such as youth-oriented campaigns on social media, may further increase exposure to PA recommendations. To maximize their impact, these interventions should be designed in alignment with adolescents’ lived experiences and cultural contexts, ensuring both relevance and uptake.

### 4.2. Limitations and Strengths

This study has several important limitations. First, the cross-sectional design does not allow for causal inferences between the variables, and the use of self-reported measures may lead to socially desirable and inaccurate responses, which could compromise the internal validity of the data. Second, the sample did not include adolescents enrolled in private schools, limiting the external validity of the findings given potential socioeconomic and behavioral differences. Third, the scope of sedentary behavior assessment was restricted to total sitting time and did not account for the variety of sedentary behaviors common in this age group. Finally, the absence of relevant contextual variables, such as family support, school practices, and access to information, may have limited a broader understanding of the factors associated with knowledge of PA guidelines.

Despite these limitations, the present study also offers important strengths. First, it addresses a relatively unexplored topic by examining adolescents’ knowledge of official PA guidelines, which remains underrepresented in the national literature, particularly among youth populations. Second, the study was conducted in Boa Vista, located in the northernmost region of Brazil, which is frequently underrepresented in population-based research. This contributes to a broader geographic and sociocultural perspective within the existing body of evidence. Third, the sample size (*n* = 1032) provided adequate statistical power and allowed for sex-stratified analyses, revealing distinct patterns of association between knowledge, PA practice, and sedentary behavior among boys and girls.

## 5. Conclusions

This study confirmed that adolescents’ knowledge of PA guidelines is low (11.7 percent) and is positively associated with meeting PA recommendations and spending less time in sedentary behavior in both sexes. These findings are consistent with international evidence, indicating that limited awareness of PA guidelines is a global public health concern. It is reasonable to suggest that more sedentary adolescents may also have lower awareness of these guidelines, reinforcing the need for targeted health promotion. Addressing this knowledge gap should be considered a priority in both school and community settings, with approaches adapted to local cultural contexts. Future research should adopt longitudinal designs, use mixed-method approaches, and investigate psychosocial and contextual factors that influence the relationship between knowledge and behavior.

## Figures and Tables

**Table 1:** General characteristics of study participants, stratified by sex (*n* = 1032).

Variables		Total	Boys	Girls
**Age**, mean (SD)		16.1 (±0.9)	16.2 (±0.5)	16.1 (±0.4)
**Skin color/race,** *n* (%)	White	218 (21.1)	104 (20.0)	114 (22.3)
	Black	159 (15.4)	101 (19.4)	58 (11.4)
	Brown	555 (53.8)	270 (51.8)	285 (55.8)
	Yellow	38 (3.7)	20 (3.8)	18 (3.5)
	Indigenous	62 (6.0)	26 (5.0)	36 (7.0)
**Monthly household income,** *n* (%)	≤2 minimum wages	614 (59.5)	278 (53.4)	336 (65.8)
	3–5 minimum wages	309 (29.9)	170 (32.6)	139 (27.2)
	≥6 minimum wages	109 (10.6)	73 (14.0)	36 (7.0)
**Head of household,** *n* (%)	Father	438 (42.4)	240 (46.1)	198 (38.7)
	Mother	594 (57.6)	281 (53.9)	313 (61.3)
**Education level of head of household,** *n* (%)	No formal education	39 (3.8)	21 (4.0)	18 (3.5)
	Elementary education	151 (14.6)	64 (12.3)	87 (17.0)
	High school	457 (44.3)	231 (44.4)	226 (44.2)
	Higher education	385 (37.3)	205 (39.3)	180 (35.2)
**Physical activity,** *n* (%)	≥60 min/day	418 (40.5)	232 (44.5)	186 (36.4)
	<60 min/day	614 (59.5)	289 (55.5)	325 (63.6)
**Sedentary behavior,** *n* (%)	≥3 h/day	917 (88.9)	433 (83.1)	484 (94.7)
	<3 h/day	115 (11.1)	88 (16.9)	27 (5.3)

Note: Values are presented as mean and standard deviation (SD) for continuous variables, and as absolute frequency (*n*) and percentage (%) for categorical variables.

**Table 2:** Crude and adjusted odds ratios (ORs) for knowledge of moderate-to-vigorous physical activity (MVPA) guidelines according to adherence to the recommendations and sedentary behavior time.

Variables		Crude		Adjusted *	
		OR (95% CI)	*p*-Value	OR (95% CI)	*p*-Value
**Boys**					
**MVPA guidelines**	≥60 min/day	*Ref.*	<0.001	*Ref.*	<0.001
	<60 min/day	14.87 (6.97–23.12)		15.76 (7.14–24.48)	
**Sedentary behavior**	≥3 h/day	*Ref.*	<0.001	*Ref.*	<0.001
	<3 h/day	2.69 (1.54–4.55)		2.00 (1.08–3.70)	
**Girls**					
**MVPA guidelines**	≥60 min/day	*Ref.*	<0.001	*Ref.*	<0.001
	<60 min/day	9.51 (4.32–15.70)		10.05 (4.43–16.67)	
**Sedentary behavior**	≥3 h/day	*Ref.*	0.01	*Ref.*	0.03
	<3 h/day	2.22 (1.45–3.45)		3.13 (1.12–8.33)	

Note: MVPA: moderate-to-vigorous physical activity; OR: odds ratio; 95% CI: 95% confidence interval; Ref.: reference category. * Model adjusted for age, household income, skin color, and head of household’s educational level.

## Data Availability

The raw data supporting this research are not publicly available due to a confidentiality agreement with the Secretaria de Estado de Educação e Desporto (SEED) of Roraima. However, qualified researchers may request access from the corresponding author upon reasonable justification.

## Abbreviations

The following abbreviations are used in this manuscript:

ICC	Intraclass correlation coefficients
IPAQ	International Physical Activity Questionnaire
MVPA	Moderate-to-vigorous physical activity
PA	Physical activity
WHO	World Health Organization
